# The Interplay Between Replacement and Retention of Histones in the Sperm Genome

**DOI:** 10.3389/fgene.2020.00780

**Published:** 2020-07-16

**Authors:** Ulises Torres-Flores, Abrahan Hernández-Hernández

**Affiliations:** Biología de Células Individuales (BIOCELIN), Laboratorio de Investigación en Patología Experimental, Hospital Infantil de México Federico Gómez, Mexico City, Mexico

**Keywords:** histones, histone replacement, histone retention, histone post-translational modifications, protamines, CTCF, epigenetic memory, transgenerational inheritance

## Abstract

The genome of eukaryotes is highly organized within the cell nucleus, this organization *per se* elicits gene regulation and favors other mechanisms like cell memory throughout histones and their post-translational modifications. In highly specialized cells, like sperm, the genome is mostly organized by protamines, yet a significant portion of it remains organized by histones. This protamine-histone-DNA organization, known as sperm epigenome, is established during spermiogenesis. Specific histones and their post-translational modifications are retained at specific genomic sites and during embryo development these sites recapitulate their histone profile that harbored in the sperm nucleus. It is known that histones are the conduit of epigenetic memory from cell to cell, hence histones in the sperm epigenome may have a role in transmitting epigenetic memory from the sperm to the embryo. However, the exact function and mechanism of histone retention remains elusive. During spermatogenesis, most of the histones that organize the genome are replaced by protamines and their retention at specific regions may be deeply intertwined with the eviction and replacement mechanism. In this review we will cover some relevant aspects of histone replacement that in turn may help us to contextualize histone retention. In the end, we focus on the architectonical protein CTCF that is, so far, the only factor that has been directly linked to the histone retention process.

## Introduction

In many organisms including mammals, spermatogenesis is a highly conserved process. Inside the seminiferous tubules in the testes, germ line cells undergo spermatogenesis to produce mature sperm. Spermatogenesis can be divided in three phases: a mitotic, a meiotic and a post-meiotic phase (Rathke et al., 2014). The meiotic phase ensures haploidization of the genome as well as an independent assortment of recombined genetic information within individual germ cells. In the post-meiotic phase, also known as spermiogenesis, cells undergo a series of morphological transformations that lead to the typical swimming torpedo-like shape of the sperm. According to their nuclear changes in shape, cells in the post-meiotic phase can be distinguished as early spermatids with round nuclei (round spermatids), intermediate spermatids with elongating nuclei (elongating spermatids) and spermatids with condensed nuclei (elongated spermatids) (Dadoune, 2003; Rathke et al., 2014).

Development of spermatids into mature sperm is a process that has been divided into 16 and 12 steps in mice and humans respectively (Ventela et al., 2002; Muciaccia et al., 2013). Throughout these steps, cells have a marked adjustment in their shape and size. Inside the nucleus, chromatin organization and compaction dramatically change during mid- to late-spermiogenesis, leading to a highly condensed genome in mature sperm (Dadoune, 2003). This is accomplished by a genome wide histone replacement by the transition nuclear proteins 1 and 2 and subsequently by the protamine 1 and 2 (TNP1, TNP2, PRM1, and PRM2; respectively) (Steger, 1999; Govin et al., 2004; Brunner et al., 2014). However, between 1–10% and 10–15% of the mouse and human genomes respectively, remain associated to histone-specific nucleosomes (Erkek et al., 2013; Jung et al., 2017; Yamaguchi et al., 2018). These retained histones are mostly on gene promoters with high content of unmethylated CpG regions and on regulatory elements, suggesting a role in the transcriptional regulation of these genes and genome organization after fertilization of an egg (Erkek et al., 2013; Jung et al., 2017).

At the stage of round spermatids there are several ongoing molecular mechanisms that may impact the organization of the sperm genome or epigenome. Thus, even though round spermatids-specific transcriptional profiles, replacement of canonic histones for testis specific and histone variants, specific histone post-translational modifications (PTMs) and formation of genomic domains may have a direct impact in the establishment of the sperm epigenome, the mechanism remains poorly understood. Histones, the architectonic protein CTCF and cohesin complexes seem to be orchestrating this mechanism (Jung et al., 2017). Thus, despite compacting most of the sperm genome in a protamine-based core, the remaining histones and architectonical regulators are shaping the sperm epigenome.

## Are Histones the Mayor Player in the Establishment of the Sperm Epigenome?

Nucleosomes with canonical histones and histone PTMs are retained in gene promoters, enhancers and super-enhancers. In addition, almost exclusively enhancers and super-enhancers also contain CTCF and cohesin complexes in mouse sperm (Jung et al., 2017). Histone PTMs and architectonical proteins profiles in the sperm epigenome are established early in the spermiogenesis process, nevertheless the interdependency of these factors is not clear. Conditional depletion of CTCF before spermiogenesis, leads to histone H2B retention defects in mature sperm (Hernandez-Hernandez et al., 2016). Although it is not known whether other histones display failures in their retention process, it seems that loss of CTCF has an impact in this process. Nonetheless, the fact that not all the histone-associated sites contain architectonical proteins suggests that histones themselves contribute to their retention process or that there are other factors that contribute to the establishment of the sperm epigenome, or both. In this regard, long non-coding RNAs have been suggested to have a role in histone modifications in mature sperm, perhaps influencing their replacement or retention processes (Zhang et al., 2017). Furthermore, the interaction of some histone variants with RNA molecules seems to stabilize a histone-protamine-based chromatin structure that is retained in mature sperm (Hoghoughi et al., 2020). Therefore, it seems that, at least in some genomic regions, histones either alone or throughout their histone-readers and effectors are the major player in the establishment of the sperm epigenome.

## Histone Replacement in the Sperm Genome

Histones are widely replaced from the sperm genome and depending of the analyzed specie, the rate of retention varies. As we will describe below, it seems that histone replacement in the sperm genome has become a more understood mechanism (broadly reviewed in Bao and Bedford, 2016; Wang et al., 2019), whereas a nucleosome retention mechanism is still at large.

## Testis-Specific Histones and Histone Post-Translational Modifications Contribute to the Nucleosome Eviction Process

**FIGURE 1:**
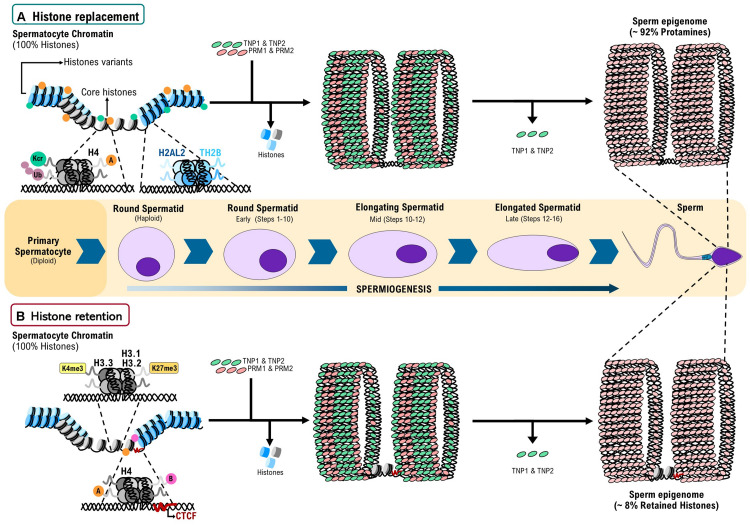
A model for histone replacement and retention during spermiogenesis in mammals. **(A)** Histone replacement: at the beginning of spermiogenesis, 100% of the genome of round spermatids is associated to nucleosomes with canonical histones (H2A, H2B, H3, H4, and H1) plus nucleosomes with testis specific histone variants (H1t, TH2A, TH2B, TH3, H2AL1, H2AL2, and H2BL1). Nucleosomes with canonical histones undergo post-translational modification (PTMs) as ubiquitination (Ub), Crotonylation (Kcr). Additionally, H4 can display Acetylation **(A)** or Butyrylation **(B)**. These PTMs and the presence of nucleosome with testis-specific histone variants facilitate histone eviction by the transition proteins 1 and 2 (TNP1 and TNP2) and subsequently by protamines 1 and 2 (PRM1 and PRM2). As result of this finely regulated process of histone replacement, 92% of the mature sperm genome is associated to protamines, in a structure called toroid. **(B)** Histone retention: genome regions with nucleosomes containing 3K4me3, H3.1K27me3, and H3.2K27me3 have a reduced intrinsic affinity for PRMs, suggesting that these PTMs are factors that promote histone retention. Additionally, Acetylation and Butyrylation in H4 may be playing a role in the processes of histone eviction and retention. Finally, CTCF bound to unmethylated DNA regions favors the positioning and retention of nucleosomes in the mouse epigenome. Around 8% of the sperm genome remains associated to histones. Partially based on Braun (2001) and Ferran Barrachina et al. (2018).

The DNA of all eukaryotes is packaged into chromatin through its association with histone proteins (Wolffe, 1998; Fan et al., 2005). There are five major classes of somatic histones: the core histones H2A, H2B, H3, and H4 and the linker histone H1 (Brunner et al., 2014). During mammalian spermiogenesis, some of these proteins are partially replaced by testis-specific histone variants. Therefore, round spermatids contain the core somatic-type histones plus the testis-specific histones and histone variants: H1T, H1T2, HILS1, TH2A, H2AL1, H2AL2, H2BL1, TH2B, TH3, and H3.3 (Figure 1; Dadoune, 2003; Govin et al., 2007; Bao and Bedford, 2016).

It has recently been demonstrated that, contrary to what was originally thought, histone to protamine exchange is not fully completed after the sperm leaves the testis. In fact, histone replacement continues during sperm movement throughout the epididymis (Yoshida et al., 2018); therefore, the full replacement of histones by PRMs should be recognized as an event that occurs in epididymis. For this process to succeed, histone variants have a key role in the eviction process because they have the potential to relax nucleosome structure and create an interaction interface required for the assembly of specific structural non-histone proteins on the DNA (Tachiwana et al., 2011; Barral et al., 2017).

Testis-specific histone variants H2AL1, H2AL2, and H2BL1 are expressed during late spermiogenesis, the stage at which the displacement of histones by PRMs takes place, suggesting their involvement in the organization of the genome (Govin et al., 2007). Indeed, recent studies have found that H2AL2 is inserted into the nucleosome core creating a flexible local structure that can be recognized by TNPs and further displaced by PRMs (Figure 1A). Accordingly, in an *H2AL2*-null mouse model, genome-wide compaction defects in sperm have been reported (Barral et al., 2017). On the other hand, TH2B partially replaces H2B in male germ cells, setting a nucleosome environment that ensures a genome-wide chromatin-to-nucleoprotamine transition (Montellier et al., 2013; Figure 1A). However, not all the eviction events produce full histone replacement. In elongating spermatids, the testis-specific nucleosomes containing H2AL2–TH2B dimers allow the invasion of nucleosomes by TNPs, permitting protamines to bind to those sites. Since the displaced histones are unable to remain as octamers, protamine–DNA and displaced transition nuclear protein–histone complexes constitute a relatively stable transitional state thereby generating small subnucleosomal structures (Barral et al., 2017), suggesting that even the histone replacement mechanism produces partial histone retention in nucleosomes with specific structure.

Histone eviction by TNPs is also influenced by histone PTMs (Braun, 2001). Acylation (i.e., acetylation and butyrylation) of H4 tails was reported to balance histone retention (Figure 1A) and removal through the acetyl lysine binding domain-containing protein (BRDT), a testis-specific double bromodomain containing chromatin remodeling factors. BRDT uses the histone hyperacetylation signal to bind chromatin and induce a “chromatin squeezing” process through a BRDT–BRDT interaction facilitating histone eviction and their replacement by TNPs (Dhar et al., 2012; Gaucher et al., 2012, Goudarzi et al., 2016). The importance of histone acetylation in the histone replacement process is also supported by studies where conditional depletion of the histone acetyl transferase Gcn5 in testis leads to an increased histone retention in sperm (Luense et al., 2019).

Additionally, it has been suggested that histone crotonylation plays a role in a second wave of histone removal in a BRDT-independent manner (Figure 1A; Liu et al., 2017). On the other hand, Ubiquitination of histones can stimulate or repress several cellular processes, as well as being associated with DNA damage responses (Huen et al., 2007; Weake and Workman, 2008). Strikingly, histone ubiquitination seems to be also crucial for the appropriate histone-to-protamine exchange process (Figure 1A), as elimination of factors responsible for this PTM during spermiogenesis (i.e., RNF8 and Piwi proteins) leads to chromatin compaction defects and abnormal histone retention in mature sperm (Lu et al., 2010; Gou et al., 2017).

H3K79 methylation is another histone PTM detected before histone eviction and correlates with the hyperacetylation of H4 that is directly associated with the eviction process in drosophila and rat, suggesting that these two PTMs act together facilitating histone eviction (Dottermusch-Heidel et al., 2014). Finally the poly ADP-ribosylation, also known as a PARylation, is a PTM produced in response to DNA strand breaks that naturally occur during spermiogenesis. These PTMs produce chromatin relaxation and allow for proper histone removal (Meyer-Ficca et al., 2011; Ihara et al., 2014).

## Transition Proteins Contribute to Histone Eviction in Mouse Spermatids

TNPs are present in many mammals including mouse (Meistrich et al., 2003) and human (Steger et al., 1998), they belong to a heterogeneous group of DNA-binding proteins that are more basic than histones but less basic than PRMs (Dadoune, 2003). TNPs are first detected in the condensing nucleus of spermatids slightly before than protamines (Heidaran et al., 1989). Whereas TNP2 is a 13 kDa protein with distinct structural domains, containing about 10% arginine, 10% lysine, and 5% cysteine (Meistrich et al., 2003; Zhao et al., 2004); TNP1 is a 6.2 KDa protein with 54 residues of amino acids, of which about 40% are arginine and lysine distributed uniformly and do not contain cysteine (Lanneau and Loir, 1982; Kleene et al., 1988; Alfonso and Kistler, 1993; Meistrich et al., 2003; Zhao et al., 2004). Some studies have suggested different functions for the TNPs like in nuclear shaping, histone removal, transcriptional repression, chromatin condensation and repair of the DNA strand breaks that transiently occur during the displacement of the nucleosomes (Caron et al., 2001; Zhao et al., 2004). Some reports indicate that TNP1 decreases the melting temperature of the DNA, releasing it from nucleosomes *in vitro* (Singh and Rao, 1988; Alfonso and Kistler, 1993; Meistrich et al., 2003). On the other hand, TNP2 is about twice the size of TNP1 and has distinct structural domains. For example the carboxyl region of the molecule is enriched in basic residues and is likely to be a major site of electrostatic DNA binding, whereas the amino terminal region has two putative zinc fingers (Baskaran and Rao, 1991; Zhao et al., 2004). The preferential binding activity of TNP2 to CpG sequences, which are often associated with promoter regions, is dependent on Zinc (Zhao et al., 2004). The time of apparition of each of these two proteins during the spermiogenesis is variable from specie to specie. In the case of mouse spermiogenesis, TNP1 and TNP2 appear in the nuclei of elongating spermatids at identical times, very close to the border between steps 12 and 13 of spermiogenesis (Alfonso and Kistler, 1993). However, some studies have reported the presence of TNP2 at the beginning of step 10 (Wu et al., 2000).

Bad sperm quality and reduced counts have been found in single or double *null* mice for *Tnp1* and *Tnp2.* Morphological analysis in these mice have revealed altered sperm morphology (Shirley et al., 2004). Neither *Tnp1* or *Tnp2* alone are haploinsufficient; in fact, mutants homozygous for either gene are fertile, however, reduction of the total *Tnp* dosage by 75% in either *Tnp1* or *Tnp2* null mice lacking one copy of the other *Tnp*, or 100% elimination of both transition protein in double *null* mutants, results in more severe abnormalities in nuclear condensation and sterility (Braun, 2001). Direct evidence of the interplay between histones and TNPs has been recently reported. Histone variant H2AL2 is crucial for the correct loading of TNPs onto the nucleosomes and for efficient PRMs assembly (Barral et al., 2017), highlighting the interplay among histones, TNPs and PRMs to achieve a proper genome compaction. Furthermore, post-translational modifications on TNPs may also contribute to the histone to protamine replacement mechanism (Gupta et al., 2015).

## From Transition Protein to Protamines

The histones that help to pack the DNA in early spermatids are evicted during spermiogenesis by other positive proteins like sperm variants histones, TNPs or PRMs. In mammals, PRMs do not evict the core canonical histones directly. Instead, this eviction is carried out by TNPs that bind to the DNA prior the PRMs (Balhorn, 2007). In mouse testis, it has been reported that expression of *Tnp1* starts slightly before than *Prm1* and *Prm2* during step 7 of spermiogenesis (Mali et al., 1989), and their newly synthetized mRNAs are stored until their translation (Steger and Balhorn, 2018). PRMs synthesis and their deposition into chromatin begins when TNP1 and TNP2 have successfully evicted the majority of the histones (Steger and Balhorn, 2018). It has been described two types of PRMs in mouse, protamine 1 and 2 (PRM1 and PRM2, respectively). The first one was identified in many vertebrates, while PRM2 it was found just in some mammals like human and mouse (Oliva, 2006). The main proposed function of these proteins are: (1) neutralize the charge of the DNA (Mali et al., 1989) aiding the compaction of the paternal genome into a 1/20 of the volume of a somatic nucleus, making the sperm nucleus highly hydrodynamic (Oliva, 2006; Steger and Balhorn, 2018), and (2) protecting the paternal genome from nucleases or environmental factors. Additionally, these proteins could confer an epigenetic mark on some regions of the sperm genome, affecting their reactivation upon fertilization (Oliva, 2006).

PRM1 is synthetized as a mature protein (Balhorn, 2007), is composed by 50 amino acid and displays three domains. A central arginine-rich domain, another domain with DNA-binding capabilities flanked on both sides by short serine residues and the last domain with threonine-containing segments with several phosphorylation sites. Furthermore, it contains cysteine residues which are able to form disulfide bridges between protamines, resulting in a tight link between them (Steger and Balhorn, 2018). On the other hand, PRM2 is synthetized as a precursor, when its processing is completed about 40% of the N-terminal of the molecule has been removed. The fully processed form of PRM2 is slightly larger than PRM1 (63 amino acids in mouse) and is the predominant form of PRM2 in the mature sperm head (Balhorn, 2007). Additionally, PRM2 displays from 50 to 70% of sequence identity with PRM1 and it is able to bind one zinc atom per molecule (Steger and Balhorn, 2018). The actual knowledge about the importance and expression time of PRMs during mouse spermatogenesis was obtained from functional studies. Deletion of either *Prm1* or *Prm2* lead to the production of sperm with abnormalities in morphology, like flagellum tightly wrapped around the head and morphological abnormalities in the nuclei. Furthermore, haploinsufficiency of any of these protamines causes infertility in mice (Cho et al., 2001). Moreover, in male chimeric mice that produced 70% of PRM2, DNA damage, morphological abnormalities in sperm and increased embryo death have been reported (Cho et al., 2003). The distribution of PRM1 overlaps with TNPs at step 10 of spermiogenesis, then progressively increased from step 11 through steps 13 or 14 and persisted through the rest of spermiogenesis. PRM2 is first detected in the spermatid nucleus at step 12, although it remains at low levels until step 14 (Zhao et al., 2004). There is evidence showing that alterations in the PRM1/PRM2 ratio, or deficiencies in zinc, or its replacement by other metals are related to infertility (Oliva, 2006; Balhorn, 2007).

## Histone Retention

In human and mouse sperm, different histone retention rates have been reported. Gatewood and cols, found a 15% of the human genome with histone retention (Gatewood et al., 1987; Oliva, 2006), whereas Hammoud and cols, reported only from 3 to 5% (Hammoud et al., 2009, 2010). Furthermore, Brykczynska and cols, reported a 10% of the genome with nucleosome presence in mature male sperm (Brykczynska et al., 2010). In the case of mouse sperm, the reported percentages vary from 8.5% to 1–10% (Jung et al., 2017; Yamaguchi et al., 2018). Furthermore, histone enrichment has been differentially detected in some sets of genes and loci, for example in the *Prm1-Prm2-Tnp2* locus (Wykes and Krawetz, 2003), in telomeres (Zalenskaya et al., 2000), in sequences around the transcription start sites (TSSs) (Brykczynska et al., 2010; Jung et al., 2017), in intergenic regions and in poor-gene regions (Jung et al., 2017; Yamaguchi et al., 2018). Some discrepancies in data have been attributed to the extraction methodology of the histones, since massive nucleosome degradation in sperm chromatin has been reported when using MNase treatment (Carone et al., 2014). To overcome this problem, a recently developed methodology, in which elimination of PRMs with nucleoplasmin prior ChIP-seq analysis, has been used to find clear localization patterns of histones in sperm chromatin, such as the enrichment of H3K4me3 in CpG-rich promoters and H3K9me3 in satellite repeats (Yamaguchi et al., 2018). In agreement with this, enrichment of H3K4me3 in TSSs of developmental genes with CpG-rich promoters have been found in independent studies (Hammoud et al., 2009; Brykczynska et al., 2010; Erkek et al., 2013; Xu and Xie, 2018; Yamaguchi et al., 2018). Another aspect that has contributed to the conflict of histone retention in different sequences of the genome, has been solved by demonstrating that replacement continues throughout the different portions of the epididymis (Yoshida et al., 2018).

The mechanism by which histones are retained during spermiogenesis is still unknown. However, some findings support a model in which histone variants H3.1, H3.2, and H3.3 are stably incorporated into nucleosomes at CpG islands (CGIs). Then PTMs on these histones, like H3K4me3 in late round spermatids, produce a global cessation of histone turnover and transcription. Furthermore, a reduced nucleosome turnover of H3K27me3 at CGIs would promote retention of canonical H3.1 or H3.2 variants. The presence of these histone variants at CG-rich in DNA could reflect a reduced intrinsic affinity to PRMs. On the other hand, a variation on this model is that transcription factors, chromatin factors/remodelers and histone H3.3 nucleosomes would continue competing for binding to CGIs during the eviction of histones by TNPs and then by PRMs, leading to regions in the sperm genome where histones are retained (Figure 1B; Erkek et al., 2013).

Additionally, it has been described that acetylation and butyrylation of H4 tails lead either to histone eviction or retention, respectively (Figures 1A,B; Goudarzi et al., 2016). Thus, it seems that histone PTMs are also important for histone retention throughout regulatory elements. Moreover, there are some genomic regions where histone variants (that usually produce eviction) lead to retention. Histone retention on pericentric heterochromatin seems to be favored by the ability of H2A.L.2 to interact with RNA (Hoghoughi et al., 2020). Thus, it seems that variations in the process of histone replacement lead rather to a retention mechanism.

There are evidences suggesting that other factors, like the transcription factors CTCF and BORIS, might be influencing histone retention in the sperm genome (Pugacheva et al., 2015). Rivero-Hinojosa and cols, found that bimodal occupancy of CTCF/BORIS and BORIS/BORIS on genomic regions associated with testis-specific transcriptional regulators was strongly linked to histone-retaining regions in mature sperm (Rivero-Hinojosa et al., 2017). Remarkably, these regions were also associated with highly expressed genes in testis and H3.3 occupancy in sperm (Erkek et al., 2013; Rivero-Hinojosa et al., 2017), suggesting a role for CTCF and BORIS in promoting high levels of transcription and histone retention. However, it is unclear if BORIS is expressed in sperm (Johnson et al., 2016), therefore these regions might be bound only by CTCF homodimers. Furthermore, despite the existence of both *Ctcf* and *Boris* knock-out mice models, only the first displays defects in chromatin organization and histone retention (Suzuki et al., 2010; Hernandez-Hernandez et al., 2016).

## CTCF as a Candidate for the Histone Retention Process During Mice Spermatogenesis

The DNA-binding factor CTCF is considered to be an architectural protein that orchestrates the three-dimensional organization of the genome with a direct impact in the fine regulation of gene expression in somatic cells. In mouse sperm, it seems that CTCF regulates chromatin organization and epigenome establishment, both of which are important for correct packaging and functionality of the paternal genome to fertilize and inherit information to the newly created embryo. This factor has been described as a zinc finger protein composed by a central zinc finger domain that binds to different sequences in the DNA molecule, while the N- and C- domains have been reported to interact with other proteins and cohesin complexes (Arzate-Mejia et al., 2018; Li et al., 2020). An approximate of ∼326,840 CTCF-binding sites in 38 different cell lines, in which the majority are ubiquitous, have been reported (Chen et al., 2012). These sites are located in intergenic regions and introns that overlap with enhancers and promoters (Arzate-Mejia et al., 2018). In mice sperm, around 23,000 CTCF binding sites overlapping with cohesin-complexes binding regions have been identified, suggesting that both proteins contribute to the 3D architecture of the sperm epigenome (Carone et al., 2014; Jung et al., 2017).

The sperm-retained histone PTMs H3K4me3, H3K4me2, and H3K27me3 have been found in promoters of early development genes, but also in regulatory elements like enhancers and super enhancers that are also occupied by CTCF (Samans et al., 2014; Jung et al., 2017). Furthermore, it has recently been shown that a small portion of CTCF sites in the genome of sperm and oocytes are maintained in preimplantation embryos. These sites are flanked by H3.3 and H2A.Z in which H3K27ac and H3K4me1 are also associated, showing that transcriptional stages between gametes and the first stages of the embryo are inherited (Jung et al., 2019). Therefore, apart from histones and histone variants, CTCF may have a role in histone retention in the sperm epigenome (Figure 1B). Concordantly, CTCF depletion at the onset of the meiotic phase during spermatogenesis, leads to mature sperm with defects in genome compaction and altered histone retention (Hernandez-Hernandez et al., 2016). However, recently it has been reported that CTCF is not present in human sperm and in consequence the 3D organization of the human sperm epigenome is not as it is in mice sperm (Chen et al., 2012, 2019; Johnson et al., 2016; Jung et al., 2019). Still, histones are retained in both epigenomes, suggesting that CTCF is not entirely (or not all) responsible for the histone retention process. Thus, more studies aiming to understand a role of CTCF or other architectonical factors in the histone retention process are still needed.

## Altered Histone Retention and Transgenerational Inheritance

It has been widely documented that histones and their PTMs (and other epigenetic factors not covered in this review) are carriers of epigenetic memory (Rathke et al., 2007; Kaufman and Rando, 2010; Jung et al., 2019; Sarkies, 2020). In the nematode *C. elegans* and in the fruit fly *D. melanogaster*, it has been shown that histones’ PTMs are responsible for inter- and transgenerational epigenetic inheritance (Skvortsova et al., 2018). In mammals, it is known that retained histones and other architectonical factors shape the sperm genome, and that this epigenome is necessary to recapitulate chromatin structure during the embryo development (van de Werken et al., 2014; Jung et al., 2017). Thus, the fact that it has been shown that proper histone retention in mammal’s sperm has a role in inter- and transgenerational epigenetic inheritance, it is not so unexpected (Siklenka et al., 2015). Strikingly environmental and toxicant factors as well as dietary exposures, can alter histone retention profiles in sperm, which may influence the epigenetically inherited traits (Terashima et al., 2015; Ben Maamar et al., 2018a, b; Skinner et al., 2018). However, what is the full impact of sperm’s altered histone retention in the offspring remains to be elucidated. Histone retention in sperm, a mechanism that is tightly intertwined with the establishment of the sperm epigenome, seems to have an impact in inter- and trans-generational epigenetic inheritance. Any alteration in the sperm epigenome seem to be enough to produce altered inherited epigenetic traits (Champroux et al., 2018; Blanco Rodriguez and Camprubi Sanchez, 2019; Cavalli and Heard, 2019; Hart and Tadros, 2019; King et al., 2019; Perez and Lehner, 2019; Shukla et al., 2019; Lewens, 2020).

## Discussion

The information presented here shows the importance of histone variants and PTMs that have to occur on histones. Firstly, for the correct displacement by transition proteins and secondly, because in some cases these chemical tags indicate which nucleosomes are going to be retained. Histone replacement by protamines is a better understood mechanism, whereas histone retention is a process that has only lately being studied. However, it seems that transcriptional programs that lead to sperm specialization and sperm epigenome establishment are codependent mechanisms that have a direct role in the histone replacement and retention processes in the mammal’s sperm.

Histones and their PTMs seem to be crucial for eviction but also for retention of histones at certain genomic regions. However, especially in regulatory elements, it seems that histones variants and PTMs are not enough to signal their retention process. Instead architectonical proteins like CTCF, may be functioning as barriers to avoid histone evection or as competitors that keep recruiting histones and thus, competing with transition proteins producing histone-containing genomic regions. Whatever the mechanism is, it seems that these retained histones play a role in transmitting memory to the embryo. Understanding how this retention is produced and its function in epigenetic memory from the sperm to the embryo may have deep impact in the current knowledge of inheritance of acquired traits throughout several generations. Furthermore, it will also shed light on how our lifestyles are shaping future generations without the need of changes in the genome as stated in the theory of evolution. Undoubtedly, more efforts to understand the mechanism of histone retention in the sperm epigenome are needed.

## Author Contributions

All authors contributed to the article and approved the submitted version.

## Conflict of Interest

The authors declare that the research was conducted in the absence of any commercial or financial relationships that could be construed as a potential conflict of interest.
